# Warts

**Published:** 1867-12

**Authors:** 


WARTS.

will disappear from the hands in a few days if red ash bark is boiled in water, strong, and the liquid applied several times every day; or if a bit of wood, an old match is dipped in aquafortis or nitric acid and rubbed on the head of the wart night and morning, it will soon disappear. Great discomfort sometimes arises from the skin at the body end of the finger nail getting shiny and then turning up; this is occasioned by the skin attaching itself to the nail and being drawn out by the nail as it grows forward, until the skin snaps by the extension, causing sometimes severe iuflammation; the best prevention is to take once a week a wooden or iron paper cutter, or even the thumb nail of the other hand and push back the skin; if a knife is used, it will make a white spot on the nail, unless done with great care; ladies sometimes prevent this by pushing back the skin with a soft cloth every time the hand is washed.

In the days of Louis XIV. the ladies of the court as a means of beautifying the skin of the face and hands and keeping it soft and velvety always washed in alcohol; perhaps pure warm water answers as good a purpose; cold water has a tendency to harden the skin, to make it coarse and certainly is not as good a cleanser as warm water.

